# Psychometrics of the Persian version of the COVID-19-related health literacy in the Iranian population

**DOI:** 10.3389/fpubh.2022.1085861

**Published:** 2023-01-10

**Authors:** Samaneh Torkian, Fatemeh Ebrahimi, Hossein Shahnazi, Roya Rashti, Mahasti Emami, Mohammad Reza Maracy

**Affiliations:** ^1^Department of Epidemiology, School of Public Health, Iran University of Medical Sciences, Tehran, Iran; ^2^Department of Epidemiology and Biostatistics, School of Health, Isfahan University of Medical Sciences, Isfahan, Iran; ^3^Department of Health Education and Promotion, School of Health Isfahan University of Medical Sciences, Isfahan, Iran; ^4^School of Public Health, Dezful University of Medical Science, Dezful, Khuzestan, Iran; ^5^Department of Health Education and Promotion, School of Health, Iran University of Medical Sciences, Tehran, Iran

**Keywords:** COVID-19, psychometric, health literacy, transcultural, validation

## Abstract

**Background:**

Since the occurrence of the COVID-19 pandemic, information dissemination has increased rapidly. Promoting health literacy is currently crucial to prepare people to respond quickly to situations, such as the COVID-19 pandemic. Due to the importance of health literacy in this critical situation, we are looking for a questionnaire to measure COVID-19 health literacy. The COVID-19 Germany Health Literacy Questionnaire (HLS-COVID-Q22) is an excellent tool, so the study aimed to create a cultural validity of this questionnaire for the Iranian population.

**Methods:**

In this validation study, 880 samples were enrolled using a convenient sampling method. The questionnaire was translated through a backward forwarding procedure. Confirmatory factor analysis (CFA) and exploratory factor analysis (EFA) were employed for Persian version validity. McDonald's omega (Ω), Cronbach's alpha, and average inter-item correlation (AIC) coefficients were assessed for reliability.

**Results:**

Using EFA on the random half sample (*n* = 440), the EFA indicated that the scale had four factors: accessing, understanding, appraising, and applying health-related information in the COVID-19 pandemic context, which explained 59.3% of the total variance. CFA was used for the sample's second part (*n* = 440) to evaluate the goodness of fit of the four-factor solution. CFA showed the model fit. All indices RMSEA = 0.067, CFI = 0.934, IFI = 0.934, PCFI = 0.772, PNFI = 0.747, and CMIN/DF = 2.972 confirmed the model fit. The convergent validity of the HLS-COVID-Q22 was confirmed. McDonald's omega and Cronbach's alpha were very good (α and Ω >0.80).

**Conclusion:**

The Persian version of the HLS-COVID-Q22 had acceptable psychometric properties and is applicable to measure COVID-19 health literacy.

## Introduction

The COVID-19 pandemic has become one of the biggest challenges for healthcare systems around the world (1). Since the occurrence of the COVID-19 pandemic, information dissemination has increased rapidly (2). In February 2020, the World Health Organization (WHO) introduced the term infodemic to describe fake news and information related to the COVID-19 pandemic. Infodemic is a “global epidemic of misinformation spreading rapidly through social media platforms and other outlets”. It has a significant impact on the COVID-19 pandemic (3).

The WHO guidelines emphasize increasing health literacy (HL) among the general public around the world to prevent the spread of infection. The concept of HL is commonly used in health communication to educate people about health-related issues. It is defined as “the degree to which individuals have the ability to acquire, process, and understand basic health” (1). HL is currently considered a set of cognitive and social skills that not only motivate but also enable individuals to acquire and use valid health-related information to promote health (4). HL is an important issue in slowing down the spread of the virus and preventing the disease. Therefore, it has been established as the global strategy to fight the COVID-19 pandemic (5).

HL may help people to understand the reasons for the recommendations and the results of their various possible actions (6). Promoting HL is more important than ever to prepare people to respond quickly to situations, such as the COVID-19 pandemic (7). Therefore, a study indicated that people who have higher levels of HL tend to be better at managing their health than those people who do not. Higher levels of HL among people are associated with increased protection, a better quality of life, less variation in health outcomes, and a more stable and equal society. People who do not have good HL cannot distinguish between fact and fiction and are more influenced by unreliable facts (2). The European HL Survey, which was conducted in eight countries, found that almost 50% of all adults have “inadequate” HL; this means that it is potentially difficult for them to understand, evaluate, and apply information to promote or protect health. Past research has shown that low HL is associated with various adverse health outcomes (3, 4). A recent study on fear of COVID-19 in medical students has shown that higher levels of HL may lower the level of fear (5). In another study, higher HL levels have shown protective effects against COVID-19-related depression (6).

Emphasis on promoting personal, community, and population HL is very important during the COVID-19 pandemic (8) which is why there is a need to explore COVID-19-related HL. However, HL is ignored or misjudged (5).

Highlight the different aspects of HL in different populations during pandemics is necessary (9). Seng et al. emphasized that “understanding the levels and determinants of HL related to the epidemic in different populations is essential for healthcare policymakers and formulating optimal and effective strategies” (7). Due to the importance of HL in this critical situation, we are looking for a questionnaire to measure COVID-19 HL. Different questionnaires have been created to measure the HL of COVID-19, and most of the studies have used COVID-19 Germany HL Questionnaire (HLS-COVID-Q22) (8) and created their own suitable questionnaire.

HLS-COVID-Q22 covers four subscales including, appraising, understanding, applying, and health-related information, while other questioners do not have these four subscales (9). In contrast, the methods of confirming the validity and reliability of some questionnaires are not enough (10). In addition, the number of questions on some scales was too many (11), which was beyond the interest of people, and finally, after many reviews, the HLS-COVID-Q22 was selected. Since the language of Iranians is different from that of Germans, as well as the different cultures of the two countries, we intend to create a culture and validity of this questionnaire for the Iranian population.

## Methods

### Study design and sample size

The current cross-sectional research was performed from 7th to 19th August 2021 on adults (equal and older than 18 years) in Iran. Inclusion criteria included age older than 18 years, having the ability to read and write, having access to the internet, and having informed consent for participation in the study. A convenient sampling method was used for online data collection. The questionnaire was prepared through Google Forms and shared its URL link through social network channels and groups (WhatsApp, Telegram, and Instagram). We included 20 participants per question in the study. Finally, 440 samples for exploratory factor analysis (EFA) and another 440 samples for evaluation confirmatory factor analysis (CFA) were enrolled (12). We used the STROBE checklist (cross-sectional studies) as a guideline for writing this article.

### Measurements

In this study, we used a two-part questionnaire. One section was related to demographic characteristics and the other section was the Persian version of COVID-19-related HL (HLS-COVID-Q22) (8). Demographic variables were age, marital status, gender, economic status, educational level, employment status, and residence.

HLS-COVID-Q22 contains 22 items with four subscales including, appraising (six items), understanding (six items), applying (five items), and health-related information (five items) in the COVID-19 pandemic context. The minimum score of this questionnaire is 22 and the maximum score is 88. The minimum and maximum average scores of this questionnaire are 1 and 4, respectively. A mean score of ≤ 2.5 indicates “inadequate HL”, a score of 2.5–3 indicates “problematic HL”, and a score of ≥3 indicates “sufficient HL” (8).

### Translation process

Before starting this study, written permission from the questionnaire's developer, Professor Orkan Okan, was obtained through email. First, the translation of the questionnaire was done into Persian by two expert independent translators. Then, the translation of the two people was compared and corrected, and a Persian questionnaire was created. In the next step, this Persian questionnaire was translated into English by a third expert translator. Two English versions were reviewed and harmonized by the research team (13).

### Face validity

Quantitative and qualitative approaches evaluated face validity. In the qualitative approach (14, 15), eight experts in health education and promotion, epidemiologist, and the instrument's development assessed the scale regarding relevancy, difficulty, and ambiguity, and all reported understandable items of the scale. In the quantitative approach, the same experts assessed the items regarding suitability by a Likert scale of five points (1 = not suitable at all, 2 = less suitable, 3 = almost suitable, 4 = suitable, and 5 = completely suitable). The following formula calculated the impact score: impact score (IS) = frequency (%) × suitability. A score of more than 1.5 was regarded as acceptable (16).

### Content validity

For content validity, quantitative and qualitative methods were used. In the qualitative approach, eight experts in health promotion and education, epidemiologist, and the instrument's development evaluated the items regarding wording and grammar, scaling, and item allocation. Then, their feedback was used to modify some items. In the quantitative method, the content validity of the scale was assessed using modified kappa statistic (K^*^) and content validity ratio (CVR).

In CVR, questions were evaluated using a three-point Likert scale (3 = essential, 2 = useful but not essential, and 1 = not essential). The formula [*n*_*e*_ – (*N*/2)]/(*N*/2) was used to calculate CVR in which “*n*_*e*_” indicates the number of experts rating the items as “essential” and “*N*” indicates the total number of experts. The Lawshe rule was applied to interpret the result. A minimum acceptable score of 0.75 was considered (17).

The chance agreement is not considered by content validity index (CVI). Therefore, K^*^ as of the combined CVI and kappa was used to correct this index.


$$\begin{array}{l} {\text{K}}^{*}=\left(\text{I}-\text{CVI}-\text{PC}\right)/\left(1-\text{PC}\right). \end{array}$$

PC indicates the chance agreement presented by the below equation:


$$\begin{array}{l} \text{PC}=\left[\text{N}!/\text{A}!\left(\text{N}-\text{A}\right)!\right]\times 0.5^{\text{N}} \end{array}$$

“*N*” indicates the number of experts and “*A*” indicates the number of experts approving the question's relevance. Regarding kappa interpretation, data of more than 0.75, 0.60–0.74, and 0.40–0.59 were excellent, good, and fair, respectively (18).

### Construct validity

Exploratory (EFA) and confirmatory factor analysis (CFA) assessed the construct validity.

### EFA

Sample size adequacy and appropriateness were checked by KMO and Bartlett's tests. The favorable KMO value is >0.70 (19). Scree plot, principal axis factoring (PAF) EFA, and ProMax rotation were used to extract latent factors. The minimum acceptance value for factor loading was determined by the CV value [CV = 5.152 ÷ √ (*n* – 2)]. CV is regarded as the minimum value that each question has to remain in a latent factor (20), where “*n*” is the sample size. Finally, in the present study, CV was calculated at 0.33. Finally, the criteria of factor extraction including eigenvalues (should be more than one), commonalities (h2, which is more than 0.3), and scree plots with factor loading were reported (21).

### CFA

The confirmatory factor analysis tested the standard HLS-COVID-Q22 structure fit. For running the model, we use the Bootstrap method. Many fit indicators are available to determine the model's goodness of fit. The fitness of the model was evaluated considering the comparative fit index (CFI >95% good and >90% acceptable), root mean square of error of approximation (RMSEA < 0.05 good and < 0.08 acceptable), parsimonious comparative fit index (PCFI >0.5 good), incremental fit index (IFI >95% good and > 90% acceptable), parsimonious normed fit index (PNFI >0.5 good), and (CMIN/DF < 3 good and < 5 acceptable) (22, 23).

### Univariate and multivariate normality and outliers

In this study, univariate distribution was assessed by the Kolmogorov–Smirnov test, skewness, and kurtosis. The cut-off values for skewness and kurtosis were considered 3 and 7, respectively (24). For checking the multivariate normality and outliers, Mardia's and Mahalanobis distance tests were used, respectively.

### Convergent and divergent validity

The divergent and convergent validity were examined by evaluating Pearson's correlations between items and factors of the HLS-COVID-Q22. We calculated Pearson's correlation between items and hypothesized factors as convergent validity and Pearson's correlation between items and other scales as divergent validity. We used a strong correlation cut-off point (r) equal to 0.70. Values of >0.70 are considered excellent correlation, and convergent correlations should always be higher than discriminant ones (25). Furthermore, construct reliability (CR), average variance extracted (AVE), and maximum shared variance (MSV) were reported. For convergent validity, CR must be greater than AVE and AVE of >0.5 (26). In divergent validity, MSV must be lower than AVE (27).

### Reliability

McDonald's omega (Ω), Cronbach's alpha, and average inter-item correlation (AIC) coefficients were applied for checking the reliability (28). According to the general rule of thumb, Cronbach's alpha equal to 0.70 and higher is considered good, 0.80 and higher is considered better, and 0.90 and higher is considered best (29). As for Cronbach's alpha, McDonald's Omega of >0.80 is regarded as good internal reliability (30). The mean inter-item correlation for some items should be from 0.20 to 0.40, which suggests that the items are reasonably homogenous and have sufficiently unique variance so that they are not identical to each other (31). SPSS, https://webpower.psychstat.org/models/kurtosis, and AMOS version 22 were applied for statistical analyses.

### Ethics

The proposal of this research was confirmed by the Ethics Committee of Isfahan University of Medical Sciences with code IR.MUI.RESEARCH.REC.1400.194. The subjects have completed a written informed consent form at the beginning of the questionnaire URL link.

## Results

### Participant characteristics

The participants' mean age was 31.09 ± 11.37 years. The majority of the participants were female (62.0%), single (56.1%), had a university education (81.1%), and were quiet in the city (94.0%). Approximately 60% of them had Fars ethnicity, 26.4% were students, and 31.9% had incomes of more than 400 dollars per month. The individuals' characteristics included in the AFA and CFA are provided separately in Table 1.

**Table 1:** Participant characteristics in the study in two exploratory (EFA) and confirmatory factor analysis (CFA) (*n* = 880).

**Variables**		**Total [No (%)]** ***N =* 880**	**EFA [No (%)]** ***N =* 440**	**CFA [No (%)]** ***N =* 440**
Age (years) (Mean ± SD)		31.09 ± 11.37	31.20 ± 11.30	30.98 ± 11.45
Gender	Male	334 (38.0)	169 (38.4)	165 (37.5)
	Female	546 (62.0)	271 (61.6)	275 (62.5)
Marital statues	Married	386 (43.9)	192 (43.6)	197 (44.1)
	Single/divorced/widow	494 (56.1)	248 (56.4)	246 (55.9)
Education	Less than high school	32 (3.6)	14 (3.2)	18 (4.1)
	High school	134 (15.2)	66 (15.0)	68 (15.5)
	University	714 (81.1)	360 (81.8)	354 (80.4)
Residence	Urban	827 (94.0)	416 (94.5)	411 (93.4)
	Rural	5 (6.0)	24 (5.5)	29 (6.6)
Ethnicity	Fars	531 (60.3)	278 (63.2)	253 (57.5)
	Lor/Lak/Bakhtiari	55 (6.3)	21 (4.8)	34 (7.7)
	Gilak/Tork/Mazani	147 (16.7)	70 (15.9)	77 (17.5)
	Other	147 (16.7)	91 (16.1)	76 (17.3)
Income means per month	< 150 dollars	160 (18.2)	79 (18.0)	81 (18.4)
	150–250 dollars	192 (21.8)	99 (22.5)	93 (21.1)
	250–400 dollars	247 (28.1)	118 (26.8)	129 (29.3)
	>400 dollars	281 (31.9)	114 (32.7)	137 (31.1)
Job	Unemployed	128 (14.5)	66 (15.0)	62 (14.1)
	Freelance job	161 (18.3)	85 (19.4)	76 (17.3)
	Employee	208 (23.6)	107 (24.3)	101 (23.0)
	Student	232 (26.4)	110 (25.0)	122 (27.7)
	Housekeeper	121 (13.8)	56 (12.7)	65 (14.7)
	Laborer	30 (3.4)	16 (3.6)	14 (3.2)

### Face validity

In the qualitative phase, the importance and wording of all questions were assessed and some modifications were made. There was no question with IS of smaller than 1.5 (Appendix Table 2).

### Content validity

Modifications were made based on the opinions of the experts. The CVI, K^*^, and CVR for each question are shown in Appendix Table 2. All values were acceptable.

### EFA

The KMO test value was 0.935, which showed sampling adequacy. Using PAF and ProMax rotation, four factors (accessing, understanding, appraising, and applying health-related information in the COVID-19 pandemic context) were extracted and explained 59.3% of the total variance (Table 2). In this model, four factors were extracted based on eigenvalues >1 (Table 2). Items 3 and 6 were removed due to cross-loading. Twenty items remained in the model because their factor load was >0.33. Eigenvalues, h2, and factor loading values are presented in Table 2.

**Table 2:** Exploratory factors extracted from items of COVID-19-health literacy (*n* = 440).

**Factors**	***Q_*n*_*. Item**	**Factor loading**	**h2**	**Eigenvalue**	**%Variance**
Factor 1 Understanding	8. Understand recommendations of authorities regarding protective measures against coronavirus infection?	0.844	0.642	9.562	45.862
	10. Understand information in the media on how to protect myself against coronavirus infection?	0.830	0.718		
	12. Understand risks of the coronavirus that I find in newspapers, magazines or on TV?	0.811	0.573		
	11. Understand risks of the coronavirus that I find on the internet?	0.760	0.635		
	9. Understand advice from family members or friends regarding protective measures against coronavirus infection?	0.599	0.587		
	7. Understand your doctor's, pharmacist's or nurse's instructions on protective measures against coronavirus infection?	0.496	0.620		
Factor 2 Appraising	16. Judge how much I am at risk for a coronavirus infection?	0.906	0.704	1.427	5.148
	14. Judge which behaviors are associated with a higher risk of coronavirus infection?	0.655	0.609		
	13. Judge if information on the coronavirus and the coronavirus pandemic in the media is reliable?	0.608	0.374		
	15. Judge what protective measures you can apply to prevent a coronavirus infection?	0.596	0.587		
	17. Judge if I have been infected with coronavirus?	0.591	0.381		
Factor 3 Accessing	2. Find information on the internet about protective behaviors that can help to prevent infection with the coronavirus?	0.914	0.777	1.301	4.634
	1. Find information about the coronavirus on the internet?	0.902	0.693		
	4. Find information on how to recognize if I have likely become infected with the coronavirus?	0.564	0.439		
	5. Find information on how to find professional help in case of coronavirus infection?	0.537	0.487		
Factor 4 Applying	20. Use information the doctor gives you to decide how to handle an infection with the coronavirus?	0.932	0.755	1.105	3.668
	19. Follow instructions from your doctor or pharmacist regarding how to handle the coronavirus situation?	0.928	0.740		
	22. To behave in a way to avoid infecting others?	0.591	0.417		
	21. Use media information to decide how to handle an infection with the coronavirus?	0.427	0.548		
	18. Decide how you can protect yourself from coronavirus infection based on information in the media?	0.393	0.583		

### CFA

To assess the best of fit of the factor structure of the 20-item COVID-19 HL scale, the chi-square goodness-of-fit test was evaluated (χ^2^ = 466.545, df = 157, *n* = 440, *p* < 0.0001) (Appendix Figure 1). Moreover, the fit of the model was examined by other indicators. All indices, such as RMSEA = 0.067, CFI = 0.934, IFI = 0.934, PCFI = 0.772, PNFI = 0.747, and CMIN/DF = 2.972, confirmed the model fit.

### Tests for normality and outliers

The Kolmogorov–Smirnov test was significant which shows data has no normality. None of the data has univariate skewness and kurtosis (skewness < 3 and kurtosis < 7). However, in the assessment of Mardia's test, multivariate skewness and kurtosis were observed (*p* < 0.001) (Appendix Table 1).

### Convergent and divergent validity

The minimum and maximum range of item-factor correlation (hypothesized and other factors) showed in Table 3. All of the item-hypothesized factor correlations were >0.70. The minimum and maximum range of convergent correlations of all factors were higher than the discriminant correlation ranges (Table 3). Appendix Table 3 shows AVE for all factors. Except for the appraising factor, AVE was >0.5, and for all factors, CR was greater than AVE, indicating acceptable convergent validity. In contrast, MSV was greater than AVE for all factors, indicating a lack of divergent validity. However, divergent validity was established using the correlation method. Overall, the convergent validity of the HLS-COVID-Q22 was confirmed.

**Table 3:** The convergent and divergent validity of Persian version of the HLS-COVID-Q22 (*n* = 440).

**Factors**	***N***	**Mean ±SD**	**Factor-items** **convergent validity Criterion 1** **(inclusive criterion)**		**Factor-items divergent validity** **Criterion 2** **(exclusive criterion)**		**Scaling fulfillment**
			**Range of factor-** **items correlations** ^a^	**Number of factor-items** **correlations**^b^	**Range of correlations** **with other factors**^c^	**Number of items** **higher correlations with** **other factors**^d^	**Number of items** **that meet criterion** **1 but not 2**
Understanding	6	13.47 ± 2.32	0.627–0.820	5/6	0.399–0.598	0/6	5/6
Appraising	5	9.41 ± 1.95	0.706–0.855	5/5	0.363–0.596	0/5	5/5
Accessing	4	9.00 ± 1.64	0.726–0.852	4/4	0.415–0.598	0/4	4/4
Applying	5	10.07 ± 1.79	0.717–0.880	5/5	0.426–0.651	0/5	5/5

^a^Pearson correlation of items-hypothesized factor.

^b^Number of item- hypothesized factor correlations that were more than 0.70.

^c^Pearson correlation of item-other factors.

^d^Number of item-other factor correlations were more than 0.70 and less than item-hypothesized factor correlations.

### Reliability

According to McDonald's omega and Cronbach's alpha, the reliability of this questionnaire was very good (α and Ω > 0.80). The details of reliability are presented in Table 4.

**Table 4:** The reliability of the Persian version of HLS-COVID-Q22 (*n* = 880).

**Factors**	**Number of** **items**	**Alpha (95% CI)**	**Omega**	**AICC (95% CI)**
Understanding	6	0.883 (0.864–0.899)	0.884	0.883 (0.871–0.895)
Appraising	5	0.829 (0.805–0.849)	0.831	0.829 (0.811–0.847)
Accessing	4	0.827 (0.804–0.851)	0.831	0.827 (0.808–0.845)
Applying	5	0.862 (0.841–0.880)	0.862	0.862 (0.847–0.876)

95% CI, 95% confidence interval (lower CI-Upper CI).

## Discussion

In this study, the four factors (understanding, appraising, applying, and health-related information in the COVID-19 pandemic context) extracted by exploratory factor analysis explained more than 59% of the variance. This study removed two items (items 3 and 6) due to cross-loading. The fitness indicators approved the construct of HLS-COVID-Q22.

The original version of HLS-COVID-Q22 (8) identified four factors with 22 items but this study identified four factors with 20 items. The four domains were the same in the two studies.

The definition of individuals' ability to deal with health information has changed. It emerged from referring to some technical skills applied, such as understanding, accessing, appraising, and applying health-related information (32). The present questionnaire has extracted all these skills in its factor analysis.

The first factor identified in the EFA was “understanding.” This item introduces approximately 45% of the total variance. The capability approach highlights the importance of “understanding” (33). This approach introduces the available resources as one of the essential preconditions for the potential to do actions. One of the most important conditions for turning such resources into the actions required to achieve the goal is the individual's understanding of these resources (34, 35). Our goal during the COVID-19 pandemic is to break the chain of transmission (36). Personal protective equipment, such as masks, disinfectants, physical distancing, and vaccines, are our resources, and people's understanding of these cases will lead to action, breaking the chain.

The potential to educate and guide individuals in the process of appraising health information is very important in the COVID-19 pandemic. Appraising ability can help people to identify information containing poor-quality arguments (false, irrelevant, or manipulative) (37). This subscale constitutes about 5% of the total variance in EFA in the current study.

The issue of access to HL is very important. With the spread of mobile phones, access to health-related information on the Internet has become more important. Older cases, as well as people who earn minimum wage or less, are less likely to have such devices (38). In addition, for people who can access digital media, there are other barriers, such as the requirement for a high level of general literacy to realize the content (39). Medical jargon and jargon, dense paragraphs, difficult formatting, and specialized language are barriers for cases that have limited HL (40).

The capability to use and process the information to guide health actions is an essential component of HL (41). A person with good health-related literacy knows when and where to search for, find, and retrieve printed data, as well as who to contact for information advice. It is very important to apply the information in making decisions at the individual and/or societal level (41).

Understanding other domains, such as appraising, applying, and information, is an appropriate tool for HL. One of the most important strengths of this questionnaire is that its questions measure both HL and electronic HL (eHL) (42). eHL and HL are crucial for decreasing the virus spread and mitigating its effect through making informed decisions regarding how to prevent and address the disease (35). HL is a social vaccine making individuals and communities able to mitigate the virus spread by using and understanding the information offered by governments and health authorities. This vaccine can be used similarly to biomedical vaccines to prevent infection from COVID-19 (43).

Despite the widespread injection of the COVID-19 vaccine in several doses and the emergence of various strains of the virus, the COVID-19 pandemic status is still present (44). This proves that other extensive and parallel interventions must perform to be able to complement the biological vaccine's effect (43). After more than 2 years of the pandemic, protocol compliance has declined. Many people, for various reasons, refuse to follow the protocols, causing the virus to spread in society and pandemics to remain dynamic (45, 46). Perhaps part of this is related to the infodemic (47). This misinformation is spread among the people and the people act far from the truth (48). HL and eHL play an important role in COVID-19 pandemic control by improving individuals' access to true health information and their ability to use it effectively (43).

### Limitations

Our most important limitation was using the self-report method leading to errors in the reports. Our sample was limited to people with Internet access and may not be representative of all Iranian people. Therefore, the study's findings should be generalized with caution. Another limitation is the lake of divergent validity based on MSV and AVE.

### Strengths of the study

This is the first study to accurately investigate the psychometric properties of the HL instrument in Iran. Strong methodology and analysis were performed in the study is an important positive point. Dealing with all aspects of validity and reliability is one of the other points.

## Conclusion

The Persian version of the HLS-COVID-Q22 scale can be an acceptable tool. Because of the characteristics, such as simple scoring, proper reliability, and validity, being answered in a short period, the questionnaire is an appropriate instrument.

## Data availability statement

The original contributions presented in the study are included in the article/Supplementary material, further inquiries can be directed to the corresponding author.

## Ethics statement

The studies involving human participants were reviewed and approved by the Ethics Committee of Isfahan University of Medical Sciences with code IR.MUI.RESEARCH.REC.1400.194. The patients/participants provided their written informed consent to participate in this study.

## Author contributions

ST, MM, and FE conceived the presented idea. ST, RR, ME, and FE contributed to the data gathering. ST and MM performed the analysis. ST and HS wrote the first draft. All authors discussed the results and contributed to the final manuscript.

## References

[1] Nutbeam D. The evolving concept of health literacy. Soc Sci Med. (2008) 67:2072–8. 10.1016/j.socscimed.2008.09.050 18952344

[2] Spring H. Health literacy and COVID-19. Health Info Libr J (2020) 37:171–2. 10.1111/hir.12322 32672399 PMC7405264

[3] Berkman ND Sheridan SL. Donahue KE Halpern DJ Viera A Crotty K. Health literacy interventions and outcomes: an updated systematic review Evidence report/technology assessment. Evid Rep Technol Assess. (2011) 199:1–941. 10.7326/0003-4819-155-2-201107190-00005 23126607 PMC4781058

[4] Sørensen K Pelikan JM Röthlin F Ganahl K Slonska Z Doyle G. Health literacy in Europe: comparative results of the European health literacy survey (HLS-EU). Eur J Public Health. (2015) 25:1053–8. 10.1093/eurpub/ckv043 25843827 PMC4668324

[5] Nguyen HT Do BN Pham KM Kim GB Dam HT Nguyen TT. Fear of COVID-19 scale—associations of its scores with health literacy and health-related behaviors among medical students. Int J Environ Res Public Health. (2020) 17:4164. 10.3390/ijerph17114164 32545240 PMC7311979

[6] Nguyen HC Nguyen MH Do BN Tran CQ Nguyen TT Pham KM. People with suspected COVID-19 symptoms were more likely depressed and had lower health-related quality of life: the potential benefit of health literacy. J Clin Med. (2020) 9:965. 10.3390/jcm9040965 32244415 PMC7231234

[7] Seng JJB Yeam CT Huang CW Tan NC Low LL. Pandemic related Health literacy–A Systematic Review of literature in COVID-19, SARS and MERS pandemics. Medrxiv. (2020). 10.1101/2020.05.07.20094227 PMC12161643 37459004

[8] Okan O Bollweg TM Berens E-M Hurrelmann K Bauer U Schaeffer D. Coronavirus-related health literacy: A cross-sectional study in adults during the COVID-19 infodemic in Germany. Int J Environ Res Public Health. (2020) 17:5503. 10.3390/ijerph17155503 32751484 PMC7432052

[9] Çeviker SA Akkaya B Akar SS. COVID-19 Health Literacy Scale Development. Int Health Trends Perspect. (2022) 2:67–79. 10.32920/ihtp.v2i1.1509

[10] Savci C Zengin N Akinci AC. Development of the Health Literacy Scale for Protection Against COVID-19. Electron J Gener Med. (2021) 18:em332. 10.29333/ejgm/11319

[11] Sanaeinasab H Saffari M Rashidi-Jahan H Rahmati F Koenig H Lin C-Y. Development and psychometric assessment of the COVID-19 health literacy scale: preliminary testing and factor structure. J Health Liter. (2022) 6:32–46. 10.22038/jhl.2021.61484.1238

[12] Comrey A Lee H. A First Course in Factor Analysis. Hoboken NJ: Taylor and Francis (2013).

[13] Ozolins U Hale S Cheng X Hyatt A Schofield P. Translation and back-translation methodology in health research–a critique. Expert Rev Pharmacoecon Outcomes Res. (2020) 20:69–77. 10.1080/14737167.2020.1734453 32089017

[14] Mazloomy Mahmoodabad SS Sadeghi R Fallahzadeh H Rezaeian M Bidaki R Khanjani N. Validity and reliability of the preventing hookah smoking (PHS) questionnaire in adolescents based on the protection motivation theory. Int J Pediatr. (2018) 6:8327–37.

[15] Al-Dhaqm A Razak S Ikuesan RA. R. Kebande V Hajar Othman S. Face validation of database forensic investigation metamodel. Infrastructures. (2021) 6:13. 10.3390/infrastructures6020013

[16] Ebadi A Zarshenas L Rakhshan M Zareiyan A Sharifnia S Mojahedi M. Principles of Scale Development in Health Science. Tehran: Jame-e-negar (2017).

[17] Ayre C Scally AJ. Critical values for Lawshe's content validity ratio: revisiting the original methods of calculation. Measur Eval Counsel Dev. (2014) 47:79–86. 10.1177/0748175613513808

[18] Cicchetti DV Sparrow SA. Developing criteria for establishing interrater reliability of specific items: applications to assessment of adaptive behavior. Am J Ment Defic. (1981) 86:127–37. 7315877

[19] Aslan H Aktürk Ü Erci B. Validity and reliability of the Turkish version of the nurse spiritual care therapeutics scale. Palliat Support Care. (2020) 18:707–12. 10.1017/S1478951520000267 32390585

[20] Goudarzian AH Sharif Nia H Harry KM Jannati Y. Assessment of the psychometric properties of the persian version of the Cardiac Self-Blame Attribution (CSBA-P) scale in patients with cardiovascular disease. OMEGA-J Death Dying. 2020:0030222820947224. 10.1177/0030222820947224 32903153

[21] Guad RM Mangantig E Low WY Taylor-Robinson AW Azzani M Sekaran SD. Development and validation of a structured survey questionnaire on knowledge, attitude, preventive practice, and treatment-seeking behaviour regarding dengue among the resident population of Sabah, Malaysia: an exploratory factor analysis. BMC Infect Dis. (2021) 21:1–11. 10.1186/s12879-021-06606-6 34465288 PMC8406825

[22] Gaskination's StatWiki CFA (2020). Available from: http://statwiki.gaskination.com/index.php?title=CFA#Measurement_Model_Invariance (accessed on April 9, 2021).

[23] Brown TA. Confirmatory Factor Analysis for Applied Research. New York, NY: Guilford publications (2015).

[24] Rozenberg R Thornhill RE Flood TA Hakim SW Lim C Schieda N. Whole-tumor quantitative apparent diffusion coefficient histogram and texture analysis to predict Gleason score upgrading in intermediate-risk 3+ 4= 7 prostate cancer. Am J Roentgenol. (2016) 206:775–82. 10.2214/AJR.15.15462 27003049

[25] Campbell DT Fiske DW. Convergent and discriminant validation by the multitrait-multimethod matrix. Psychol Bull. (1959) 56:81. 10.1037/h0046016 13634291

[26] Shrestha N. Factor analysis as a tool for survey analysis. Am J Appl Math Stat. (2021) 9:4–11. 10.12691/ajams-9-1-215330692

[27] Afework T Wondimagegnehu A Alemayehu N Kantelhardt EJ Addissie A. Validity and reliability of the Amharic version of supportive care needs survey-short form 34 among cancer patients in Ethiopia. BMC Health Serv Res. (2021) 21:1–10. 10.1186/s12913-021-06512-2 34020635 PMC8138921

[28] Ercan I Yazici B Sigirli D Ediz B Kan I. Examining Cronbach alpha, theta, omega reliability coefficients according to sample size. J Modern Appl Stat Methods. (2007) 6:27. 10.22237/jmasm/1177993560

[29] Punyasettro S Wangwongwiroj T Yasri P. An Assessment Tool for Measuring Learners' Self-Efficacy. Psychol Educ J. (2021) 58:104–10. 10.17762/pae.v58i4.450030615610

[30] Feißt M Hennigs A Heil J Moosbrugger H Kelava A Stolpner I. Refining scores based on patient reported outcomes–statistical and medical perspectives. BMC Med Res Methodol. (2019) 19:1–9. 10.1186/s12874-019-0806-9 31366326 PMC6670170

[31] Denis F Rouached I Siu-Paredes F Delpierre A Amador G El-Hage W. Psychometric properties of the schizophrenia oral health profile: preliminary results. Int J Environ Res Public Health. (2021) 18:9090. 10.3390/ijerph18179090 34501679 PMC8430897

[32] Diviani N. On the centrality of information appraisal in health literacy research. HLRP: Health Liter Res Pract. (2019) 3:e21–e4. 10.3928/24748307-20181214-01 31294303 PMC6608914

[33] Robeyns I. Capability approach. Handbook of Economics and Ethics. Cheltenham, United Kingdom: Edward Elgar Publishing (2009).

[34] Diener E. The Science of Well-Being: The Collected Works of Ed Diener. Berlin, Germany: Springer (2009).

[35] Falk Erhag H Lagerlöf Nilsson U Rydberg Sterner T Skoog I. A Multidisciplinary Approach to Capability in Age and Ageing. Berlin, Germany: Springer Nature (2022). p. 70.

[36] WHO. Breaking the chain of COVID-19 transmission: the key role of Camp-wise Rapid Investigation and Response Teams. (2020). Available online at: https://www.who.int/bangladesh/news/detail/10-11-2020-breaking-the-chain-of-covid-19-transmission-the-key-role-of-camp-wise-rapid-investigation-and-response-teams (accessed November 10, 2020).

[37] Rubinelli S Ort A Zanini C Fiordelli M Diviani N. Strengthening critical health literacy for health information appraisal: an approach from argumentation theory. Int J Environ Res Public Health. (2021) 18:6764. 10.3390/ijerph18136764 34201894 PMC8269373

[38] Smith B Magnani JW. New technologies, new disparities: the intersection of electronic health and digital health literacy. Int J Cardiol. (2019) 292:280–2. 10.1016/j.ijcard.2019.05.066 31171391 PMC6660987

[39] Janiak E Rhodes E Foster AM. Translating access into utilization: lessons from the design and evaluation of a health insurance Web site to promote reproductive health care for young women in Massachusetts. Contraception. (2013) 88:684–90. 10.1016/j.contraception.2013.09.004 24120249

[40] Thies K Anderson D Cramer B. Lack of adoption of a mobile app to support patient self-management of diabetes and hypertension in a federally qualified health center: interview analysis of staff and patients in a failed randomized trial. JMIR Human Factors. (2017) 4:e7709. 10.2196/humanfactors.7709 28974481 PMC5645643

[41] Liu C Wang D Liu C Jiang J Wang X Chen H. What is the meaning of health literacy? A systematic review and qualitative synthesis. Family Med Commun Health. (2020) 8:e000351. 10.1136/fmch-2020-000351 32414834 PMC7239702

[42] Norman CD Skinner HA. eHEALS: the eHealth literacy scale. J Med Internet Res. (2006) 8:e507. 10.2196/jmir.8.4.e27 17213046 PMC1794004

[43] Okan O Messer M Levin-Zamir D Paakkari L Sørensen K. Health literacy as a social vaccine in the COVID-19 pandemic. Health Promot Int. (2022). 10.1093/heapro/daab197 35022721 PMC8807235

[44] Khandia R Singhal S Alqahtani T Kamal MA Nahed A Nainu F. Emergence of SARS-CoV-2 Omicron (B. 1.1. 529) variant, salient features, high global health concerns and strategies to counter it amid ongoing COVID-19 pandemic. Environ Res. (2022) 2022:112816. 10.1016/j.envres.2022.112816 35093310 PMC8798788

[45] Cartaud A Quesque F Coello Y. Wearing a face mask against Covid-19 results in a reduction of social distancing. PLoS ONE. (2020) 15:e0243023. 10.1371/journal.pone.0243023 33284812 PMC7721169

[46] Abeya SG Barkesa SB Sadi CG Gemeda DD Muleta FY Tolera AF. Adherence to COVID-19 preventive measures and associated factors in Oromia regional state of Ethiopia. PLoS ONE. (2021) 16:e0257373. 10.1371/journal.pone.0257373 34669723 PMC8528333

[47] Siebenhaar KU Köther AK Alpers GW. Dealing with the COVID-19 infodemic: Distress by information, information avoidance, and compliance with preventive measures. Front Psychol. (2020) 2020:2981. 10.3389/fpsyg.2020.567905 33224060 PMC7674611

[48] Patel MP Kute VB Agarwal SK. behalf of COVID O. “Infodemic” COVID 19: more pandemic than the virus. Indian J Nephrol. (2020) 30:188. 10.4103/ijn.IJN_216_20 33013069 PMC7470201

