# Supplementary material for: Psychometrics of the Persian version of the COVID-19-related health literacy in the Iranian population
Source: Front Public Health. 2023 Jan 10;10:1085861. doi: 10.3389/fpubh.2022.1085861 (PMC9873230; doi:10.3389/fpubh.2022.1085861)
Supplement: Supplementary file 1 [file Data_Sheet_1.docx]

Table 1. Univariate and multivariate skewness and kurtosis (n = 440)

| Variables | Skewness | SE_ Skewness | | Z_ Skewness | | Kurtosis | SE_ Kurtosis | | Z_ Kurtosis |
| --- | --- | --- | --- | --- | --- | --- | --- | --- | --- |
| Q1 | -0.635 | 0.116 | | -5.461 | | 0.730 | 0.232 | | 3.145 |
| Q2 | -0.626 | 0.116 | | -5.376 | | 0.774 | 0.232 | | 3.334 |
| Q4 | -0.440 | 0.116 | | -3.782 | | 0.065 | 0.232 | | 0.280 |
| Q5 | -0.372 | 0.116 | | -3.195 | | -0.058 | 0.232 | | -0.248 |
| Q7 | -0.582 | 0.116 | | -4.997 | | 0.336 | 0.232 | | 1.449 |
| Q8 | -0.728 | 0.116 | | -6.257 | | 0.564 | 0.232 | | 2.427 |
| Q9 | -0.501 | 0.116 | | -4.301 | | 0.694 | 0.232 | | 2.986 |
| Q10 | -0.545 | 0.116 | | -4.679 | | 0.965 | 0.232 | | 4.157 |
| Q11 | -0.477 | 0.116 | | -4.095 | | 0.810 | 0.232 | | 3.489 |
| Q12 | -0.528 | 0.116 | | -4.534 | | 0.368 | 0.232 | | 1.585 |
| Q13 | -0.063 | 0.116 | | -0.543 | | -0.576 | 0.232 | | -2.479 |
| Q14 | -0.283 | 0.116 | | -2.433 | | -0.105 | 0.232 | | -0.450 |
| Q15 | -0.460 | 0.116 | | -3.953 | | 0.351 | 0.232 | | 1.509 |
| Q16 | -0.287 | 0.116 | | -2.463 | | -0.083 | 0.232 | | -0.358 |
| Q17 | -0.175 | 0.116 | | -1.503 | | -0.287 | 0.232 | | -1.235 |
| Q18 | -0.533 | 0.116 | | -4.580 | | 0.437 | 0.232 | | 1.882 |
| Q19 | -0.422 | 0.116 | | -3.627 | | 0.503 | 0.232 | | 2.167 |
| Q20 | -0.574 | 0.116 | | -4.935 | | 1.009 | 0.232 | | 4.345 |
| Q21 | -0.511 | 0.116 | | -4.388 | | 0.962 | 0.232 | | 4.142 |
| Q22 | -0.530 | 0.116 | | -4.558 | | 0.093 | 0.232 | | 0.401 |
| Mardia's multivariate skewness and kurtosis | | | | | | | | | |
|  | | | b | | z | | | p-value | |
| Skewness | | | 81.23 | | 5956.90 | | | <0.001 | |
| Kurtosis | | | 656.23 | | 76.45 | | | <0.001 | |

Table 2. Impact Score, Content Validity Ratio and Adjusted Kappa in evaluating the Face, Content and Construct Validity of the HLS-COVID-Q22 Scale

| Row | Items | Content validity | | | Face validity | Interpretation  Items |
| --- | --- | --- | --- | --- | --- | --- |
|  |  | CVR | K* | Interpretation  K* | IS |  |
| 1 | Find information about the coronavirus on the internet? | 0.75 | 1 | Excellent | 4.87 | Remained |
| 2 | Find information on the internet about protective behaviors that can help to prevent infection with the coronavirus? | 1 | 1 | Excellent | 4.87 | Remained |
| 3 | Find information in newspapers, magazines and on TV about behaviors that can help to prevent infection with the coronavirus? | 0.75 | 1 | Excellent | 4 | Remained |
| 4 | Find information on how to recognize if I have likely become infected with the coronavirus? | 1 | 1 | Excellent | 5 | Remained |
| 5 | Find information on how to find professional help in case of coronavirus infection | 1 | 1 | Excellent | 4.87 | Remained |
| 6 | Find information on how I much I am at risk for being infected with the coronavirus? | 0.75 | 1 | Excellent | 5 | Remained |
| 7 | Understand your doctor’s, pharmacist’s or nurse’s instructions on protective measures against coronavirus infection? | 0.75 | 1 | Excellent | 4.87 | Remained |
| 8 | Understand recommendations of authorities regarding protective measures against coronavirus infection? | 1 | 1 | Excellent | 4 | Remained |
| 9 | Understand advice from family members or friends regarding protective measures against coronavirus infection? | 0.75 | 0.87 | Excellent | 2.37 | Remained |
| 10 | Understand information in the media on how to protect myself against coronavirus infection? | 1 | 1 | Excellent | 4.87 | Remained |
| 11 | Understand risks of the coronavirus that I find on the internet? | 1 | 1 | Excellent | 4.87 | Remained |
| 12 | Understand risks of the coronavirus that I find in newspapers, magazines or on TV? | 0.75 | 0.87 | Excellent | 4.15 | Remained |
| 13 | Judge if information on the coronavirus and the coronavirus epidemic in the media is reliable? | 1 | 1 | Excellent | 4.87 | Remained |
| 14 | Judge which behaviors are associated with a higher risk of coronavirus infection? | 1 | 1 | Excellent | 5 | Remained |
| 15 | Judge what protective measures you can apply to prevent a coronavirus infection? | 1 | 1 | Excellent | 5 | Remained |
| 16 | Judge how much I am at risk for a coronavirus infection? | 1 | 1 | Excellent | 5 | Remained |
| 17 | Judge if I have been infected with coronavirus? | 1 | 1 | Excellent | 5 | Remained |
| 18 | Decide how you can protect yourself from coronavirus infection based on information in the media? | 1 | 1 | Excellent | 4.87 | Remained |
| 19 | Follow instructions from your doctor or pharmacist regarding how to handle the coronavirus situation? | 1 | 0.87 | Excellent | 4.15 | Remained |
| 20 | Use information the doctor gives you to decide how to handle an infection with the coronavirus? | 1 | 1 | Excellent | 4.15 | Remained |
| 21 | Use media information to decide how to handle an infection with the coronavirus? | 1 | 1 | Excellent | 4.15 | Remained |
| 22 | to behave in a way to avoid infecting others? | 1 | 1 | Excellent | 5 | Remained |

Table 3. Convergent validity, divergent validity, and construct reliability of the HLS-COVID-Q22 (n = 440)

| Dimension | CR | AVE | MSV |
| --- | --- | --- | --- |
| Understanding | 0.861 | 0.511 | 0.674 |
| Appraising | 0.848 | 0.484 | 0.606 |
| Accessing | 0.861 | 0.556 | 0.674 |
| Applying | 0.838 | 0.513 | 0.558 |

CR: construct reliability, AVE: Average Variance Extracted, MSV: Maximum Shared Squared Variance


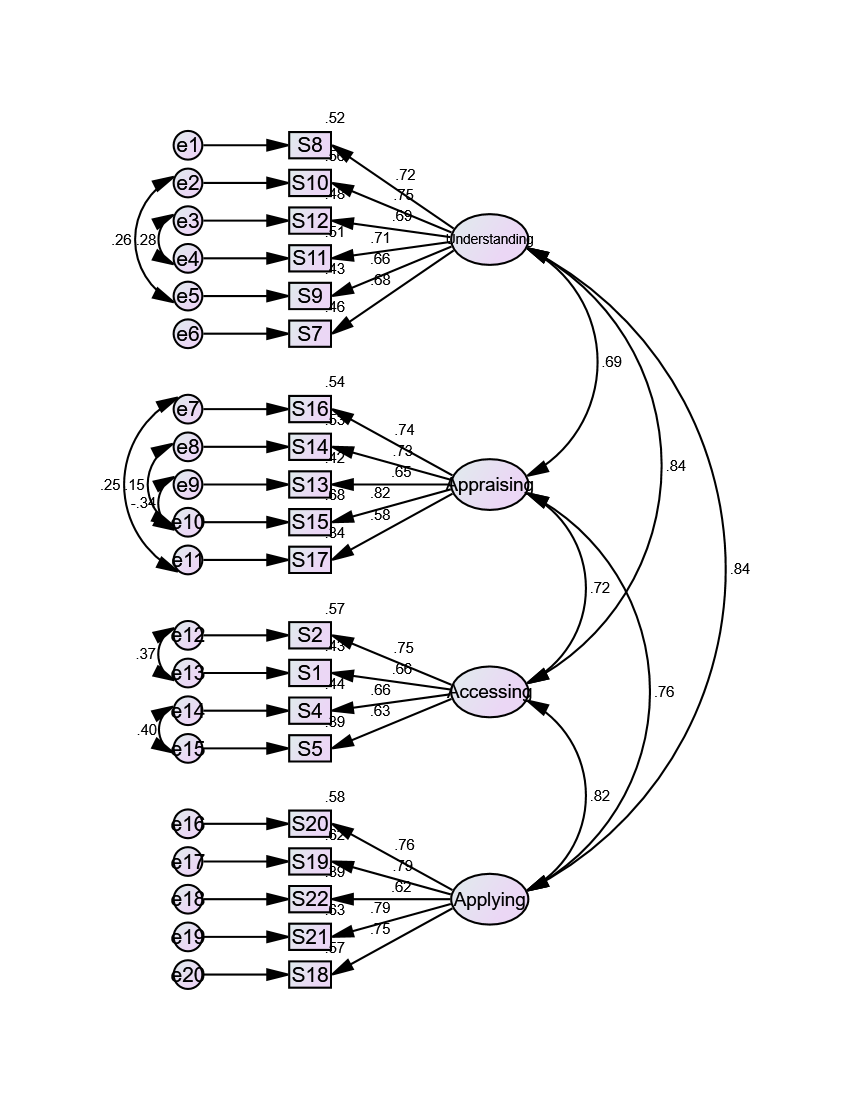


Figure 1. The results obtained from CFA of HLS-COVID-Q20
